# Normal Numbers of Stem Cell Memory T Cells Despite Strongly Reduced Naive T Cells Support Intact Memory T Cell Compartment in Ataxia Telangiectasia

**DOI:** 10.3389/fimmu.2021.686333

**Published:** 2021-06-24

**Authors:** Thomas J. Weitering, Janine E. Melsen, Monique M. van Ostaijen-ten Dam, Corry M. R. Weemaes, Marco W. Schilham, Mirjam van der Burg

**Affiliations:** ^1^ Laboratory for Pediatric Immunology, Department of Pediatrics, Willem-Alexander Children’s Hospital, Leiden University Medical Center, Leiden, Netherlands; ^2^ Department of Pediatrics, Radboudumc Amalia Children’s Hospital, Radboud University Medical Center, Nijmegen, Netherlands

**Keywords:** Ataxia Telangiectasia, cellular immunity, stem cell memory T cells, spectral flow cytometry, recent thymic emigrants

## Abstract

Ataxia Telangiectasia (AT) is a rare inherited disorder characterized by progressive cerebellar ataxia, chromosomal instability, cancer susceptibility and immunodeficiency. AT is caused by mutations in the ATM gene, which is involved in multiple processes linked to DNA double strand break repair. Immunologically, ATM mutations lead to hampered V(D)J recombination and consequently reduced numbers of naive B and T cells. In addition, class switch recombination is disturbed resulting in antibody deficiency causing common, mostly sinopulmonary, bacterial infections. Yet, AT patients in general have no clinical T cell associated infections and numbers of memory T cells are usually normal. In this study we investigated the naive and memory T cell compartment in five patients with classical AT and compared them with five healthy controls using a 24-color antibody panel and spectral flow cytometry. Multidimensional analysis of CD4 and CD8 TCRαβ^+^ cells revealed that early naive T cell populations, i.e. CD4^+^CD31^+^ recent thymic emigrants and CD8^+^CCR7^++^CD45RA^++^ T cells, were strongly reduced in AT patients. However, we identified normal numbers of stem cell memory T cells expressing CD95, which are antigen-experienced T cells that can persist for decades because of their self-renewal capacity. We hypothesize that the presence of stem cell memory T cells explains why AT patients have an intact memory T cell compartment. In line with this novel finding, memory T cells of AT patients were normal in number and expressed chemokine receptors, activating and inhibitory receptors in comparable percentages as controls. Comparing memory T cell phenotypes by Boolean gating revealed similar diversity indices in AT compared to controls. We conclude that AT patients have a fully developed memory T cell compartment despite strongly reduced naive T cells. This could be explained by the presence of normal numbers of stem cell memory T cells in the naive T cell compartment, which support the maintenance of the memory T cells. The identification of stem cell memory T cells *via* our spectral flow cytometric approach is highly relevant for better understanding of T cell immunity in AT. Moreover, it provides possibilities for further research on this recently identified T cell population in other inborn errors of immunity.

## Introduction

Ataxia Telangiectasia (AT) is a rare autosomal recessive disease characterized by cerebellar ataxia and progressive neurodegeneration, telangiectasia of the skin and eye, susceptibility to ionizing radiation (IR), predisposition to cancer, mostly of lymphoid origin and immunodeficiency (1, 2). The laboratory findings associated with immunodeficiency in AT are highly variable, even in family members carrying the same mutation (3, 4). Around two-thirds of patients present with abnormalities in the immune system, most commonly T and B cell lymphopenia and immunoglobulin IgG2, IgG4 and IgA deficiency (1, 5). Some patients present with elevated IgM and can be wrongly diagnosed as Hyper-IgM syndrome (6). AT has an estimated prevalence of <1-9/100,000 and an incidence of between 1 in 20,000 -100,000 live births (1).

The classification of AT is based on absence of the affected Ataxia Telangiectasia Mutated (ATM) protein and/or its kinase activity: patients without ATM protein or kinase activity present as classic AT with (early) childhood onset. Patients with variant AT have ATM protein with residual activity resulting in a milder form of the disease, often with adult onset (1). ATM is often dubbed the master regulator of DNA double stranded break (DSB) repair. ATM is recruited to DSBs, where it is activated and subsequently phosphorylates its downstream signaling targets (1, 2). Through its diverse and vast substrate library ATM influences many cellular processes, most importantly DNA DSB repair, cell cycle regulation, apoptosis and cellular senescence, but also mitochondrial and oxidative stress responses (2). In the adaptive immune system, ATM is involved in generation of the B-cell receptor (BCR) and T-cell receptor (TCR) repertoire *via* its supportive role in V(D)J recombination (7, 8). In addition, ATM facilitates Class Switch Recombination (CSR) in B cells (9, 10). Both V(D)J recombination and CSR are mediated by DNA DSBs.

The immunodeficiency in AT patients most often presents as humoral immunodeficiency with common, mostly sinopulmonary, bacterial infections (5). This has been attributed to the disturbed generation of naive B cells, resulting in low numbers and reduced immune repertoire diversity in naive B cells leading to suboptimal B-cell responses and antibody deficiency because of suboptimal CSR (2, 11). It is remarkable that despite these deficits, also in T cell numbers, even the early onset Classic AT patients do, in general, not suffer from severe systemic viral disease or opportunistic diseases (5), such as Pneumocystis jirovecii pneumonia, which are characteristic for cellular immunodeficiencies.

AT patients have a profound lack of naive cells and a reduced, skewed TCR beta repertoire when analyzed in total CD4 and CD8 T cells due to a disturbed generation in the thymus (11–13). The thymic tissue is usually hypoplastic, with reduced lymphocytes and lacking Hassall corpuscles (14). The number of CD31^+^ recent thymic emigrants (RTEs) (15) is strongly decreased as are the TCR excision circles (TRECs) (12, 16). In part of the AT patients the measured TREC levels are very low, resulting in identification of AT patients as incidental finding in newborn screening for Severe Combined Immunodeficiencies (SCID) (17), which is based on quantification of TRECs on dried blood spots (18). However, despite the strongly reduced counts of the naive T cell compartment, the memory T cell compartment seems to be normal in size (11, 12).

The aim of this study is to gain more insight into the T cell immunity in AT. We aim to tackle the question of why the reduced number of total, and severely reduced number of naive T cells, does not lead to opportunistic diseases in the vast majority of AT patients. In this article, we present our findings obtained through 24-marker spectral flow cytometry of T cells in classical AT followed by dimensionality reduction analyses. By separately, hierarchically analyzing the naive and memory T cells, we demonstrated that the memory T cell compartment of AT patients is not only normal in size, but also has an immunophenotype fully comparable with healthy controls. Detailed analysis of the naive CCR7^+^CD45RA^+^ population revealed that naive T cells with a stem cell memory phenotype (CD95^+^) are present in similar numbers as in control samples. The combined results suggest that the limited naive T cell population is sufficient to build up and maintain a functional memory T cell population.

## Materials & Methods

### Patients

Peripheral blood samples and clinical data were collected from 5 classic AT patients, who are under follow up at the Radboud University Medical Center (**Table S1**). Ethical approval was obtained from the Ethical Committee Arnhem/Nijmegen with protocol registration number 2011/304. Written informed consent was obtained from all patients. Five healthy control subjects were also included in our study (B17.001). Leukocyte subsets (absolute leukocyte counts and differential) were measured by using a Sysmex hematology analyzer and Trucount tubes (Becton Dickinson (BD), Franklin Lakes, NJ, USA) with a mixture of antibodies specific for lymphocyte subsets in freshly collected blood samples. Antibodies included CD45 (2D1, BD), CD3 (SK7; BD), CD19 (J4-119, Beckman Coulter, Brea, CA, USA), CD16 (3G8, BD) and CD56(C5.9, DAKO, Glostrup, Denmark). Samples were measured on a BD CANTO flow cytometer and analyzed with BD DIVA software. Peripheral blood mononuclear cells (PBMCs) were isolated using Ficoll density gradient centrifugation and cryo-stored before use.

### Spectral Flow Cytometry

**Figure 1:**
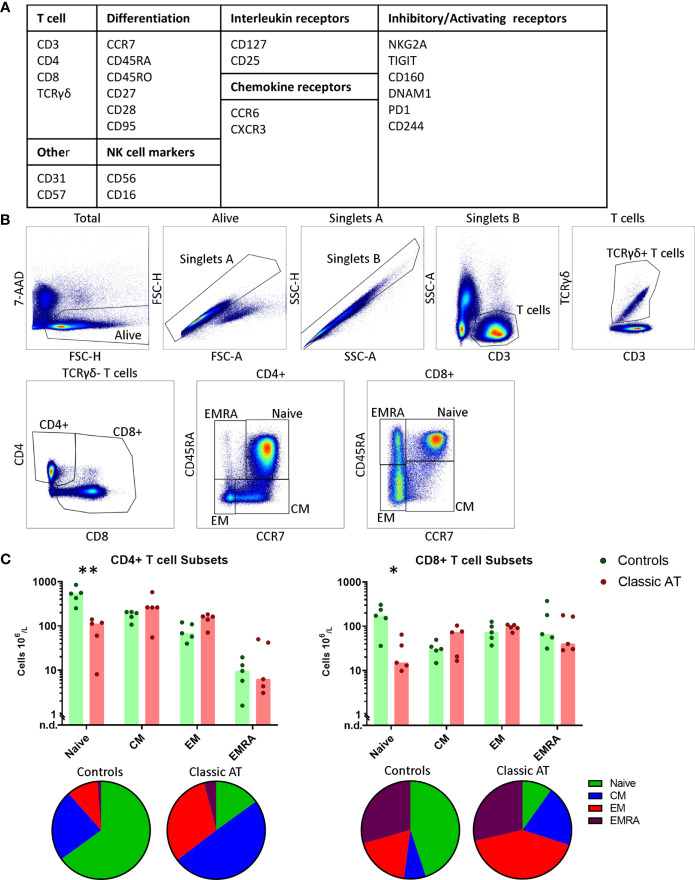
Gating Strategy and T-cell composition in AT patients and controls. **(A)** Overview of the included markers in the antibody panel for spectral flowcytometry grouped per category**. (B)** Gating strategy for T cells. From left to right: live cells; singlets based on forward scatter; singlets based on side scatter; CD3^+^ and low side scatter T cells; TCRγδ^+^ T cells; CD4^+^ and CD8^+^ T cells; and finally naive, central memory (CM), effector memory (EM) and terminally differentiated effector memory cell (EMRA) gates within the CD4 and CD8 T cells based on CCR7 and CD45RA expression. **(C)** Absolute counts of naive, CM, EM and EMRA T cells within the CD4 (left) and CD8 (right) T cells, visualized in bar graphs (median) and individual closed circles of the classic AT (red) and controls (green). Bottom: Pie charts of the percentages of naive, CM, EM and EMRA T cells within the classic AT patients and controls. * means P < 0.05, and ** means P ≤ 0.01.

PBMCs were phenotyped using a 24-marker spectral flow cytometry panel (**Figure 1A** and **Table S2**). Frozen PBMCs were thawed using AIM-V medium (Thermo Fisher Scientific, Waltham, Massachusetts, United States) supplemented with 100 IU/mL Pencillin, 100 mg/mL Streptomycin and 20% heat inactivated fetal calf serum (thawing medium) supplemented with DNAse (1600 IU per mL (Thermo Fisher Scientific)) and incubated for 5 minutes at 37°C. PBMCs were washed twice and subsequently incubated for one hour at 37°C in thawing medium. Cells were washed twice in FACS buffer (PBS supplemented with 0.5% BSA, 2 mM EDTA and 0.02% NaN3) and counted. One million PBMCs per sample were incubated in the dark for 45 minutes at room temperature with fluorochrome-conjugated antibodies (**Table S2**) and Brilliant Stain buffer plus (BD) in FACS buffer. Next, the cells were washed twice in FACS buffer and resuspended in a final volume of 100 µL. 7-AAD was added to the cell suspension 5 minutes prior to sample acquisition. Flow cytometry was performed with a 5-Laser Cytek Aurora flow cytometer (Aurora 5L-1, Cytek^®^ Biosciences Inc, Fremont, California, United States) at the Flow cytometry Core Facility (FCF) of the Leiden University Medical Center (LUMC). The measurements and spectral unmixing were performed using the Spectroflo^®^Software (Cytek Biosciences Inc.).

### Data Analysis

Data analysis was performed on the unmixed FCS files using the cytometry analysis platform OMIQ (Omiq, Inc, Santa Clara, CA, USA). Data were manually compensated and arcsinh transformed, as previously described (19). Technical variation was minimized by performing the complete experiment on one day. Live single cells were gated based on forward scatters, side scatters and absence of 7-AAD (**Figure 1B**). To remove anomalous events, flowAI (20) was applied based on the flow rate and dynamic range of the fluorochrome parameters. A maximum of 4.1% events of total live single cells was removed. Next, the T cells were selected by gating for low side scatter CD3^+^ events (**Figure 1B**) and further separated in TCRαβ^+^ and TCRγδ^+^ cells (**Figure 1B**). CD4^+^ and CD8^+^ T cells were gated from TCRαβ cells and further divided into naive and non-naive (memory) cells (**Figure 1B**). These latter four populations were used as input for either Uniform Manifold Approximation and Projection (UMAP) (21) or optimized Stochastic Neighbor Embedding (Opt-SNE) (22). Downsampling was performed for the CD4 and CD8 memory T cell subsets of all samples to include an equal number of cells per sample. For the CD4 and CD8 naive T cells only control samples were downsampled to equal numbers while all naive cells from patients were included due to the low numbers. Both opt-SNE and UMAP were calculated based on all fluorochrome parameters, except 7-AAD. Standard settings were used, except the minimum distance was set at 0.4 for UMAP. The inversed Simpson index (1/Dominance) (23) was calculated based on the frequencies of distinct phenotypes using the abdiv package in R(v3.6.3, R Foundation for Statistical Computing, Vienna, Austria). To identify the different phenotypes, Boolean gating was performed by using FlowType in R, based on the following markers: CD27, CD28, DNAM1, CD57, CD244, CD56, PD1, CD160, TIGIT, NKG2A, CCR6, CXCR3, CCR7 and CD25.

### Statistical Analysis

Visualizations and statistical analyses were performed in either Graphpad Prism v8.4.2 (GraphPad Software, California, United States) or R. Continuous outcome measures were compared between two groups using multiple unpaired-t testing. The Holm-Sidak correction was applied to correct for multiple testing. Statistical significance was set as an adjusted p-value of <0.05.

## Results

### Naive T Cells Are Severely Diminished in AT But Memory T Cells Are Not

In this study we included 5 classic AT patients (age range: 15-24 years), of which 2 did not express ATM protein, while the other 3 expressed ATM protein without kinase activity (**Table S1**). The clinical characteristics, including immunoglobulin levels, infectious episodes, intravenous immunoglobulin (IVIG) substitution treatment and vaccination responses of the AT patients are also summarized in **Table S1**. Of the five patients, only AT1 received IVIG substitution. Notably, patients AT1 and AT5, those who completely lack ATM protein, were more susceptible to bacterial infections. The age range of the 5 healthy controls was between 9-59 years. The absolute numbers of T cells of all samples, both CD4 and CD8, were within normal range (**Table S1**, **Figure S1**). The only exception was AT sample 5, where total and CD8^+^ T cell numbers were above the normal reference range. A high variety in absolute numbers of TCRγδ^+^ T cells was observed in both control and patient samples **(** **Figure S1** and **Table S1** **)**. The absolute numbers of B cells and NK cells were normal in the patients, except for patient 1 with decreased B cells. The subpopulations of NK cells CD56^bright^, CD56^dim^CD16^+^ and CD56^dim^CD16^-^ were equally distributed between patients and controls (**Table S1** and **Figure S2** **).**

For the spectral flow cytometry, PBMCs were stained with 24 monoclonal antibodies (**Figure 1A** and **Table S2**). First, live single CD3^+^ T cells events were gated (**Figure 1B** **)**. Based on the CD4 and CD8 staining of the CD3^+^TCRγδ^-^ cells, the CD4^+^ T cells were gated allowing for CD4+ T cells expressing low levels of CD8. CD8^+^ T cells were gated to include CD4^+^CD8^+^ T cells (**Figure 1B**). CD4^+^ and CD8^+^ T cell populations were individually subsampled for downstream analysis and will hereafter be referred to as the CD4 and the CD8 T cells. Within both the CD4 and CD8 populations of T cells, we further defined the naive, central memory (CM), effector memory (EM) and terminally differentiated effector memory (EMRA) T cells based on CD45RA and CCR7 expression (**Figure 1B**). In line with previous literature, patients had severely diminished naive CD4 and CD8 T cell numbers (**Figure 1C**), whereas the sizes of the memory subsets were comparable to the controls (**Figure 1C**). This also resulted in a proportional shift towards the memory cells at the expense of the naive T cells (**Figure 1C**; pie charts).

### Atypical Dominant CD45RAdimCCR7^+^ Populations Identified in Two AT Patients

**Figure 2:**
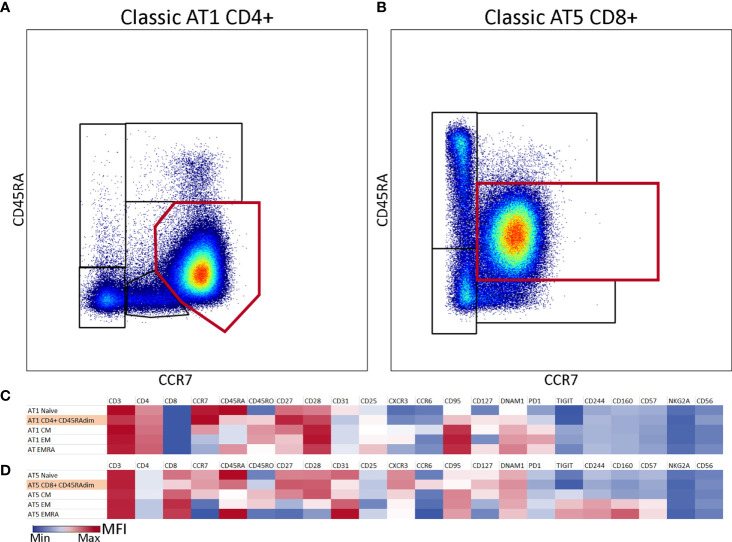
Atypical CD45RA^dim^CCR7^+^ population in two samples of classic AT patients. **(A)** CD4 T cells from classic AT1 showing a large atypical population (86%) which is visualized by the red gate on top of the differentiation subset gates. **(B)** CD8 T cells from classic AT5 showing a large atypical population (76%) which is visualized by the red rectangular gate on top of the differentiation subset gates. **(C)** Heatmap of marker intensities [Mean Fluorescent Intensities (MFI; MIN (blue) – MAX (red)] of the CD4^+^ atypical population and the differentiation subsets from sample AT1. **(D)** Similar to C, but for the CD8^+^ atypical population and the differentiation subsets from sample AT5.

A large proportion of the T cells from patients AT1 and AT5 was characterized by an abnormal phenotype based on the expression pattern of CD45RA and CCR7. This population was present in addition to the classical naive, CM, EM and EMRA T cells. In AT1 this population comprised 86% of CD4 T cells, and in AT5 this population represented 76% of the CD8 T cells (**Figures 2A, B** **)**. The fact that the highest total CD4 T cell number of all patients was observed in AT1 (1026 x 10^6^ cells/L) and the highest total CD8 T cell number was observed in AT5 (1523 x 10^6^ cells/L) highlights the abundancy of these cells **(** **Table S1**). Further exploration of the phenotype of these populations led us to conclude that the majority of the markers on both the CD4 and CD8 CD45RA^dim^ populations were homogenously expressed **(** **Figures S3** and **S4**). A heterogenous expression of CD31, CD95 and CXCR3 on a minor fraction of the cells was observed (**Figures S3** and **S4** **).** The CD45RA^dim^CCR7^+^CD27^+^CD28^+^ phenotype combined with the absence of exhaustion or activation markers suggests that these cells are phenotypically positioned between the naive and CM population **(** **Figures 2C, D** **).** While the presence of a dominant population may be of relevance for further characterization of AT patients, we decided to exclude these abnormal populations from further analysis of the CD4 and CD8 T cell populations.

### Normal Numbers of Stem Cell Memory Cells in the Naive T Cell Compartment of AT Patients

**Figure 3:**
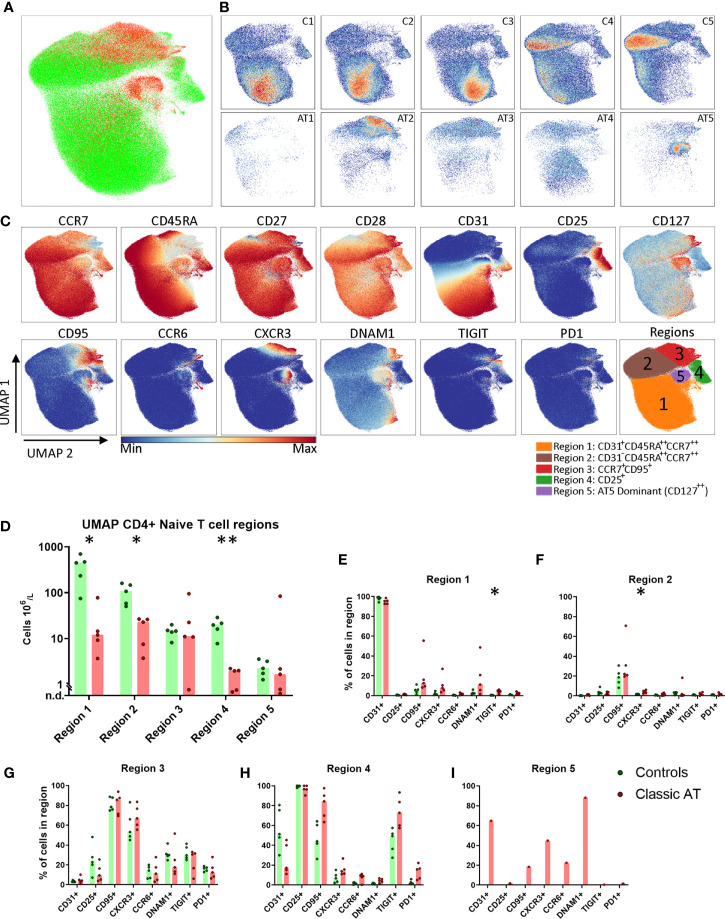
UMAP of naive CD4 T cells highlights lack of naive CD31^+^ Recent Thymic Emigrants and CD25^+^ naive T regulatory, but comparable numbers of stem cell memory T cells. **(A)** UMAP plot of CD4 naive T cells, showing the cells of the classic AT patients (red; 72829 cells) positioned on top of controls (green; 431765 cells). **(B)** Individual UMAP density plots for each sample, on the top row controls and on the bottom row classic AT patients. **(C)** Fluorochrome intensities superimposed on the UMAP embedding for a subset of markers (All markers shown in **Figures S5A, B**). Based on these marker intensities, the overlay in A and individual sample plots in B, we defined 5 regions, as shown in the bottom-right plot: CD31^+^CD45RA^++^CCR7^++^ (Region 1), CD31^-^CD45RA^++^CCR7^++^ (Region 2), CCR7^+^CD95^+^ (Region 3), CD25^+^ (Region 4) and CD127^++^ cells (Region 5). **(D)** Calculated absolute blood cell counts based on events within each region visualized in bar graphs (median) and individual dots of the classic AT (red) and controls (green). **(E–I)** Bars graphs (median) and individual closed circles of the Classic AT (red) and controls (green) showing the percentage of Boolean gating defined positive cells in each region. * means P < 0.05, and ** means P ≤ 0.01.

Because the numbers of naive T cells in AT patients were severely diminished, the composition and phenotype of the remaining naive T cells were further explored. First, we characterized the naive CD4 T cells. To visualize the high-dimensional data in 2 dimensions, the dimensionality reduction algorithm UMAP was applied, based on all fluorochrome parameters (**Figure 3A**). UMAP has the advantage that it can reveal potential differentiation patterns, since cells that are more similar to each other are closer together and dissimilar cells are further away from each other.

Although the total number of CD4 naive T cells included in this analysis from patients was much lower (72829) compared to controls (431765), it was evident that the patient cells were not scattered but restricted to only certain regions of the UMAP embedding (**Figure 3A**). This indicates that certain cell populations are almost completely lacking in AT patients, while others are still present. Density plots of the individual samples indicated that there were still clear differences in distribution of naive T cells between the individual control and individual patient samples (**Figure 3B**). A selection of markers of which the expression is colored on the UMAP of all samples is shown in **Figure 3C**. The complete set of markers of controls and AT patients is shown in **Figures S5A** and **S5B**, respectively.

To quantify these observations, we defined 5 regions in the UMAP embedding, guided by the density plots and the expression of the parameters: CD31^+^CD45RA^++^CCR7^++^ (Region 1), CD31^-^CD45RA^++^CCR7^++^ (Region 2), CCR7^+^CD95^+^ (Region 3), CD25^+^ (Region 4) and CD127^++^ cells (Region 5) (**Figure 3C**). As previously reported, the CD31^+^ recent thymic emigrants in region 1 were present in significantly lower numbers in AT patients compared to healthy controls (medians: 12 x 10^6^ cells/L vs. 448 x 10^6^ cells/L; **Figure 3D** **)**. Besides CCR7 and CD45RA, only CD27 and CD28 were expressed on these cells (**Figures 3C, E** **)**. The CD31^+^ cells were also present in lower numbers in the control samples C4 and C5, which are from older individuals, aged 37 and 59 years (**Figure 3B**). Region 2 contained naive cells that were no longer expressing CD31 but were sharing (high) expression of CCR7, CD45RA, CD27 and CD28 with cells from region 1 (**Figure 3C**). 20% of the cells from patients and controls expressed CD95 (**Figure 3F**). The absolute number of CD31^-^ cells from region 2 was also significantly lower in the AT group compared to controls (**Figure 3D**). The CCR7^+^ cells in region 3 were further characterized by a lower expression of CD45RA and CCR7 than cells in region 1 and by expression of CD95 and CXCR3, markers associated with stem cell memory cells (**Figures 3C, G** **)**. DNAM1, TIGIT and PD1 were expressed on 10-40% of the cells (**Figure 3G**). Interestingly, the absolute cell numbers in patients were comparable to controls (**Figure 3D**), except in one patient (AT1, **Figure 3D**). The proportion of cells expressing each of the markers was also comparable in region 3 between patients and controls, indicating that there were no major differences in phenotypes (**Figure 3G**).

Region 4 was identified by a uniform expression of CD25 and contained the regulatory T cell (Treg) population. The absolute number of Treg cells was significantly higher in control samples than in the patients (median: 20 x 10^6^/L vs. 2 x 10^6^/L; **Figure 3D**). In the patients there were markedly fewer CD25^+^ cells expressing CD31 compared to controls (median: 17% vs. 51%) (**Figure 3H**). Moreover, higher percentages of cells expressing CD95 in AT patients (median: 44% vs. 84%) and TIGIT (median: 49% vs. 73%) were observed, indicating that, like in the conventional naive T cells, there was a relative skewing towards cells that had encountered antigen within the Tregs (**Figures 3C, E, H** **)** (24). Region 5 was dominated by cells from 1 patient sample, AT5 (**Figures 3B, D** **)**. These cells expressed CD127, CD31, DNAM1, CD27, CD28 and partly CD95, CXCR3 and CCR6 (**Figures 3C, I** **)**.

**Figure 4:**
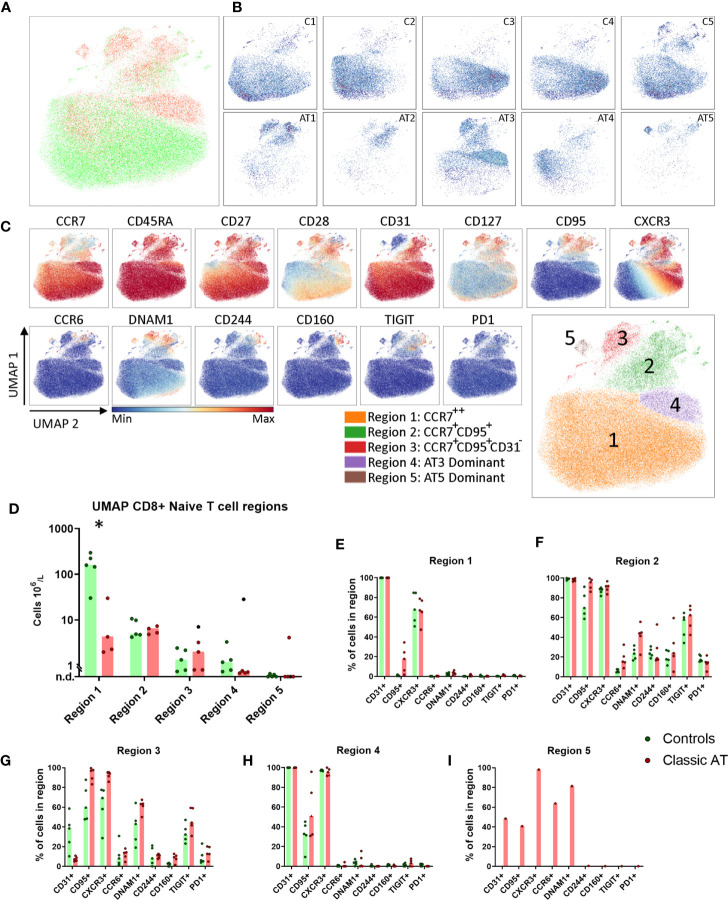
UMAP of naive CD8 T cells highlights reduced CD31^+^ cells, but comparable numbers of stem cell memory T cells. **(A)** UMAP plot of CD8 naive T cells, showing the cells of the classic AT patients (red; 24769 cells) positioned on top of controls (green; 61945 cells). **(B)** Individual UMAP density plots for each sample, on the top row controls and on the bottom row Classic AT. The density plots indicate clear differences in cell distributions between individual samples in both groups. **(C)** Fluorochrome intensities superimposed on the UMAP embedding for a subset of markers (All markers shown in **Figures S6A, B**). Based on these marker intensities, the overlay in A and individual sample plots in B, we defined 5 regions, as shown in the bottom-right plot: CCR7^++^ (Region 1), CCR7^+^CD95^+^ (Region 2), CCR7^+^CD95^+^CD31^-^ (Region 3), AT3 Dominant (Region 4) and AT5 Dominant, or exclusive (Region 5). **(D)** Absolute counts of cells within each region, visualized in bars graphs (median) and individual closed circles of the classic AT (red) and controls (green). **(E–I)** Bar graphs (median) and individual closed circles of the classic AT (red) and controls (green) showing the percentage of Boolean gating defined positive cells in each region. * means P < 0.05.

We applied the same analysis to the naive CD8 T cells. Again, a higher number of cells were analyzed from the controls (61945) compared to the AT patients (24769). The patient and control sample distribution over the UMAP embedding revealed both control- and patient-specific regions (**Figures 4A, B** **)**. A selection of markers colored on the UMAP is shown in **Figure 4C**. The complete set of markers of controls and AT patients is shown in **Figures S6A** and **S6B** respectively. Five regions were assigned in the UMAP of the naive CD8 T cells (**Figure 4C** **);**. Region 1 contained CCR7^++^CD45RA^++^ cells that expressed CD27, CD28 and CD31 [which is not a marker for recent thymic emigrants in CD8 cells (25)] and were present in higher numbers in controls than in patients (160 x 10^6^ cells/L vs. 4 x 10^6^ cells/L) (**Figures 4B–E**). Region 2 was characterized by lower CCR7 expression and high percentages of CD95 and CXCR3 expressing cells, two markers associated with stem cell memory T cells. DNAM1 and TIGIT were expressed on 40-60% and CD244, CD160, PD1 on 10-40% of the cells (**Figure 4F**). The absolute numbers of the cells in region 2 in patients and controls were equal (**Figure 4D**). Region 3 was phenotypically and quantitatively similar to region 2 except that cells lacked CD31 expression (**Figures 4D, G** **)**. This suggests that the stem cell memory cell compartment has a normal size in AT patients. In regions 1, 2 and 3 CD95, CXCR3, CCR6, TIGIT, DNAM1, CD244, CD160 and PD1 were not differentially expressed between the patients and controls (**Figures 4E–G**).

Regions 4 was dominated by cells from patient AT3 (**Figure 4B**). Cells in region 4 had a phenotype similar to cells in region 1, except that all cells were CXCR3^+^ and more cells were CD95^+^ (**Figures 4E, H** **)**. Region 5 only contained cells from patient AT5 and was characterized by CXCR3 expression and a heterogenous expression of CD31, CD95, CCR6 and DNAM1 (**Figure 4I**). Together, these findings demonstrated that based on CCR7, CD45RA, CD31, CD95, CXCR3, CCR6, DNAM1 and TIGIT multiple naive T cell subsets were identified. Remarkably, despite reduction in total naive CD8 T cells, T cells with a stem cell memory phenotype were present in normal numbers in AT patients.

### Memory T Cell Phenotypes in AT Patients Are as Heterogenous as in Controls

**Figure 5:**
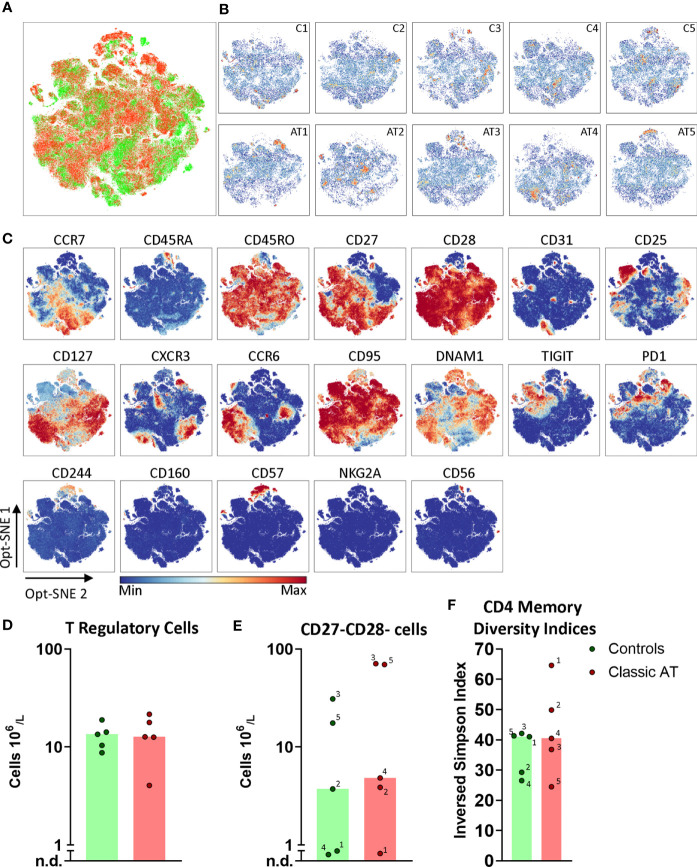
Opt-SNE and phenotypical diversity indices in the CD4 memory compartment reveals similar heterogeneity in classic AT and controls. **(A)** CD4 memory T cells Opt-SNE plot, showing the cells of the classic AT (red) patients positioned on top of controls (green). **(B)** Individual Opt-SNE density plots for each sample, on the top row controls and on the bottom row classic AT. **(C)** Fluorochrome intensities superimposed on the Opt-SNE embedding for a subset of markers (All markers shown in **Figures S7A, B**). **(D)** Bar graphs (median) and individual closed circles of the classic AT (red) and controls (green) showing the calculated absolute blood cell count of Boolean gating defined cells: CD25^+^CD127^low^ regulatory T cells. **(E)** Idem for CD27^-^CD28^-^ T cells. **(F)** The diversity or inversed Simpson index of the CD4 memory T cells is shown as bars (median) and individual closed circles of the classic AT (red) and controls (green). The index was calculated based on the Boolean gating of the following markers: CD28, CD27, DNAM1, CD57, CD244, CD56, PD1, CD160, TIGIT, NKG2A, CCR6, CXCR3, CCR7 and CD25. In total 16384 possible phenotypes were analyzed, of which 1518 were detected in the CD4 memory T cells.

Because there were no differences in absolute numbers of CM, EM and EMRA cells between patients and controls, the memory T cells were analyzed as total non-naive T cells and visualized in opt-SNE embeddings. In contrast to the naive CD4 T cells, the memory CD4 T cells from both the patient and control groups were evenly scattered (**Figure 5A**). The density plots depicting all individual samples illustrated that the density of memory T cells was equally distributed per sample (**Figure 5B** **).** The advantage of opt-SNE is its power to accentuate phenotypically identical cells by grouping of the cells. However, no distinct clusters were revealed by the embedding. The CM, EM and EMRA populations were not clearly separated based on CCR7 and CD45RA (**Figure 5C**, complete set of Opt-SNE in **S7A, B**). We did observe a CD25^+^CD127^low^ Treg population and a CD27^-^CD28^-^ cluster at the top of the embedding (**Figure 5C**). The Treg population was quantified in all samples and absolute numbers of cells were equal in patients and controls (**Figure 5D**), illustrating that also within the Treg population, there was only a relative lack of naive cells. This CD27^-^CD28^-^ cluster was further defined by CD244, DNAM1 and a heterogenous expression of CD45RA, CD45RO and CD57, indicating that memory subsets are better defined based on CD27 and CD28, rather than CD45RA and CCR7. The highest frequency of CD27^-^CD28^-^ CD4 T cells was observed in the controls C2, C3 and C5 that were seropositive for CMV, but also in AT3 (CMV^+^) and AT5 (CMV serostatus unknown) (**Table S1** and **Figure 5E**).

Since no distinct additional clusters were revealed, we decided to calculate a diversity index, based on the Boolean gating of all chemokine receptors, interleukin receptors, activating and inhibitory receptors to reflect the phenotypical heterogeneity in the CD4 memory compartment. In total more than 16000 possible phenotypes were defined, of which 1518 were detected. The inversed Simpson index, reflecting diversity, is based on the distribution of these phenotypes and revealed that the diversity in controls (median 41.0) and AT patients (median 40.5) was comparable (**Figure 5F**). The most frequent phenotype in all samples was CD27^+^CD28^+^DNAM1^+^CD57^-^ with absence of other activating and inhibitory receptors (**Figure S8A**). Together, these findings indicated that the CD4 memory T cell compartment of AT patients is as heterogenous as in the control samples.

**Figure 6:**
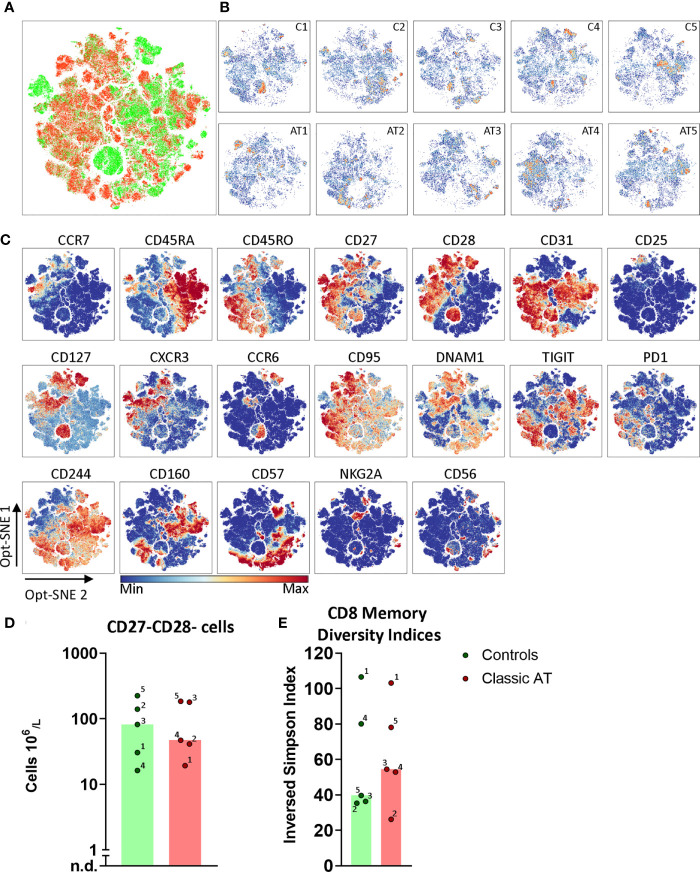
Opt-SNE and phenotypical diversity indices in the CD8 memory compartment reveals similar heterogeneity in classic AT and controls. **(A)** CD8 memory T cells Opt-SNE plot, showing the cells of the classic AT (red) patients positioned on top of controls (green). **(B)** Individual Opt-SNE density plots for each sample, on the top row controls and on the bottom row classic AT. **(C)** Fluorochrome intensities superimposed on the Opt-SNE embedding for a subset of markers (All markers shown in **Figures S8A, B**). **(D)** Bars (median) and individual closed circles of the classic AT (red) and controls (green) showing the calculated absolute blood cell count of Boolean gating defined CD27^-^CD28^-^ T cells. **(E)** The diversity or inversed Simpson index of the CD8 memory T cells is shown as bars (median) and individual closed circles of the classic AT (red) and controls (green) The index was calculated based on the Boolean gating of the following markers: CD28, CD27, DNAM1, CD57, CD244, CD56, PD1, CD160, TIGIT, NKG2A, CCR6, CXCR3, CCR7 and CD25. In total 16384 possible phenotypes were analyzed, of which 3396 were present in the CD8 memory T cells.

Also for the CD8 memory T cells we applied opt-SNE to reveal phenotypically identical groups of cells. Again, no obvious differences between distribution of memory cells in patient and control samples was observed (**Figures 6A, B** **;** complete set in **S9A–B**), except that we identified multiple sample specific groups of cells. For instance, a CD127^++^CCR6^+/-^CD28^++^ subset was only observed in C1, C3 and C4 (**Figures 6B, C** **)**. In addition, multiple sample specific clusters were observed in the CD28^-^ and/or CD27^-^ memory CD8 T cells, which were further defined based on CD56, CD57 and all activating and inhibitory receptors, such as TIGIT, NKG2A and CD160. The CD27^-^CD28^-^ memory CD8 T cells were present at higher numbers in C2, C3 and C5 (all CMV^+^ serostatus), but also in AT3 (CMV^+^) and AT5 (CMV serostatus unknown) (**Figure 6D**) (26, 27). To quantify all possible phenotypes based on the chemokine receptors, interleukin receptors and activating/inhibitory receptors, we again applied Boolean gating which revealed 3396 different phenotypes. The diversity index showed that the median diversity was comparable between controls and patients (**Figure 6E** **).** The CMV seropositive C2, C3, and C5 had the lowest diversity, as explained by the high frequency (9.8%, 14.4% and 9.5% respectively) of the most frequent phenotype (CD27^-^CD28^-^CD57^+^; **Figure S8A**). This is line with the high absolute number of the CD27^-^CD28^-^ CD8 T cells (**Figure 6D**). These findings indicate that the diversity analysis is able to pick up imbalances in the memory compartment. The spread in diversity indices of samples of patients and controls was surprisingly similar in both the CD4 and the CD8 memory T cells (**Figures 5F**, **6E**). In conclusion, the diversity of the memory T cells in AT patients and controls was comparable.

## Discussion

In this study, we analyzed the T cell compartment of classic AT patients in order to better understand why the severely reduced naive T cell compartment does not prevent the formation and maintenance of memory T cells and consequently does not lead to opportunistic infections in the vast majority of patients. Until now it was known that the naive T cells in AT patients were severely decreased in number, but the memory T cells were present in normal numbers. By investigating the expression of a broad panel of markers associated with activation, differentiation and ‘exhaustion’ of T cells, we present a detailed overview of both the naive and the memory T cell compartment. Our main conclusion based on this in-depth analysis is that the composition of the memory compartment is phenotypically as heterogeneous as that of healthy controls. This is in line with the observation that AT patients do not have clinical symptoms which can be attributed to T cell dysfunction. Regarding the reduced naive T cell compartment, we confirmed that CD4^+^ recent thymic emigrants were reduced in absolute number as well as in frequency. However, we identified in this naive compartment a population of cells that is present in equal numbers in AT patients and controls, which is a novel observation in AT. These cells express CD95 and CXCR3 which is consistent with the phenotype of stem cell memory cells, and we hypothesize that these stem cells are responsible for proper maintenance of the memory T cell compartment (28–33).

In 2 of the 5 samples of AT patients, AT1 and AT5, we identified a dominant and homogenous atypical Tcell population based on CCR7 and CD45RA expression. In preliminary analyses using opt-SNE or UMAP, these cells are clearly separated from the normal cells. It would be interesting to follow up the development of such populations in longitudinal samples and combine the analysis with cellular or molecular Tcell receptor repertoire studies. These cells could be transiently present as a result of dysregulation, or they may be oligoclonal or even pre-malignant cells (34). Interestingly, these two patients (AT1 and AT5) both completely lacked ATM protein and were the most susceptible to bacterial infections within our small cohort (**Table S1**). However, studies in mice show a more severe phenotype for kinase-dead ATM, i.e. embryonic lethality (35). The respective effects of ATM protein loss or isolated loss of kinase activity on both the severity of immunodeficiency and the development of these atypical T cell expansions in human patients remains to be further studied in a larger patient cohort.

It has been reported that AT patients have a severely reduced naive T cell population in peripheral blood, containing low TRECs, and few CD4 T cells expressing CD31 (12). Here we confirmed these observations in the CD4 compartment. In this regard the AT patients are reminiscent of aged healthy controls, who also have a reduction in these population. However, the lack of naive CD31^+^ T cells in AT patients appears more pronounced than in our oldest control sample (59 years). Similarly, within the naive CD4^+^CD25^+^ Treg population (**Figure 3C**; Region 4), which was mostly lacking in AT patients, there is a relative decrease in the proportion of cells expressing the naive marker CD31. In contrast, the Treg cells within the memory compartment, that are expressing CD95 and TIGIT, are still present in AT patients in normal numbers. The observed lack of the early (CD31^+^) naive conventional T cells appears to be recapitulated in the composition of the Treg cells.

A surprising, novel finding was that a T cell subpopulation within the naive CCR7^+^CD45RA^+^ gate expressed CD95 and CXCR3. The CD95^+^ CD8 T cells also expressed and TIGIT (and partially PD1) on a proportion of the cells. This population was present in equal numbers in both controls and AT patients, in contrast to the earlier naive T cell subpopulations, which were severely reduced in AT. The combination of CD95 and CXCR3 has previously been reported to be expressed on stem cell memory Tcells (30). More recently, the existence of CD8 stem cell memory progenitor cells, expressing TIGIT and PD1 giving rise to T cells with an ‘exhausted’ phenotype has been postulated (36). Stem cell memory cells are antigen-experienced and because of their self-renewal capacity they can persist for decades (30). We hypothesize that the stem cell memory population, consisting of more than one type of stem cell memory cells, explains why these patients have an intact memory T-cell compartment.

Detailed analysis of the memory T cell compartment in AT did not reveal a more ‘exhausted’ phenotype in AT compared to the controls analyzed, which could be expected in AT patients based on similarity with another DNA repair disorder, the Nijmegen Breakage syndrome (37). By assigning diversity indices to the various samples, it became apparent that the phenotypical variability was comparable between samples in the control and classic AT group. By assigning diversity indices, it became apparent that the phenotypical diversities of classic AT patients were within the range of the controls. This suggests that the memory T cells of AT patients and controls were equally well equipped to respond to antigenic challenges.

The observation that the memory T cells are indistinguishable from healthy controls, is in line with the clinical observation that AT patients, in general, do not seem to have immunological complications, ascribed to T cell defects. Clearly, this study has shown that the limited number of naive T cells in AT is sufficient to mount adequate memory responses, and that the presence of stem cell memory T cells may explain how these memory T cells are maintained. This is highly relevant for better understanding of T cell immunity in AT. Moreover, it provides possibilities for further research on this recently identified T cell population in other inborn errors of immunity.

## Data Availability Statement

The raw data supporting the conclusions of this article will be made available by the authors, without undue reservation.

## Ethics Statement

The studies involving human participants were reviewed and approved by the Ethical Committee Arnhem/Nijmegen, The Netherlands. Written informed consent to participate in this study was provided by the participants’ legal guardian/next of kin.

## Author Contributions

TW, CW, MS, and MB contributed to conception and design of the study. TW and MO-tD performed the experiments. TW, JM, and MO-tD performed the analysis. TW, JM, MS, and MB wrote the manuscript. All authors contributed to the article and approved the submitted version.

## Funding

This study was supported with a research grant from Action for A-T (grant WAKZ 4519). JM was supported by fellowships from the Leiden University Medical Center, the graduate program of Nederlandse Organisatie voor Wetenschappelijk Onderzoek, and a grant from Stichting Zeldzame Ziekten Fonds (SCID project).

## Conflict of Interest

The authors declare that the research was conducted in the absence of any commercial or financial relationships that could be construed as a potential conflict of interest.
