# Supplementary material for: Normal Numbers of Stem Cell Memory T Cells Despite Strongly Reduced Naive T Cells Support Intact Memory T Cell Compartment in Ataxia Telangiectasia
Source: Front Immunol. 2021 Jun 24;12:686333. doi: 10.3389/fimmu.2021.686333 (PMC8264762; doi:10.3389/fimmu.2021.686333)
Supplement: Supplementary file 1 [file Table_1.docx]

Supplementary Material

Supplemental Table 1: Characteristics of AT patients and healthy controls

| **Patient** | **Age** | **ATM Protein** | **ATM Kinase Activity** | **T cells** | **CD4^+^ T Cells** | **CD8^+^ T Cells** | **TCRγδ^+^ T Cells** | **NK Cells** | **NK Cells: CD56^bright^** | **NK Cells: CD56^dim^ CD16^+^** | **NK Cells: CD56^dim^ CD16^-^** | **B cells** | **Immunoglobulin Deficiencies** | **IVIG Therapy** | **CMV Serostatus** | **Infectious Disease(s)** |
| --- | --- | --- | --- | --- | --- | --- | --- | --- | --- | --- | --- | --- | --- | --- | --- | --- |
| **Control 1** | 9 | NA | NA | 1550 | 1082 | 400 | 48 (L) | 210 | 12 | 175 | 23 | 230 | NA | No | Negative | NA |
| **Control 2** | 16 | NA | NA | 1883 | 862 | 498 | 488 (H) | 164 | 16 | 117 | 31 | 140 | NA | No | Positive | NA |
| **Control 3** | 25 | NA | NA | 1521 | 906 | 492 | 96 | 198 | 11 | 173 | 14 | 214 | NA | No | Positive | NA |
| **Control 4** | 37 | NA | NA | 843 | 577 | 236 | 16 (L) | 229 | 13 | 150 | 66 | 410 | NA | No | Negative | NA |
| **Control 5** | 59 | NA | NA | 1091 | 531 | 533 | 12 (L) | 165 | 9 | 139 | 17 | 208 | NA | No | Positive | NA |
| **Classic AT1** | 15 | Absent | - | 1200 | 1027 | 152 | 14 (L) | 170 | 17 | 78 | 75 | 40 (L) | Total IgG and subsets, IgA | Yes | Unknown | Frequent Bacterial Airway Infections |
| **Classic AT2** | 15 | Present | - | 810 | 554 | 169 | 77 | 220 | 22 | 81 | 117 | 120 | IgG2 and IgG4 | No | Unknown | No. Good vaccination responses. |
| **Classic AT3** | 21 | Present | - | 1280 | 548 | 390 | 332 (H) | 250 | 11 | 206 | 34 | 150 | IgA | No | Positive | No. Good vaccination responses. |
| **Classic AT4** | 23 | Present | - | 1300 | 872 | 276 | 135 (H) | 250 | 17 | 114 | 119 | 260 | None | No | Unknown | No. |
| **Classic AT5** | 24 | Absent | - | 2710 (H) | 592 | 1524 (H) | 33 | 450 | 19 | 292 | 139 | 180 | Mild IgG2 | No | Unknown | Multiple bacterial pneumonias. 1x Pseudomonas urosepsis. |
| **Reference values [1]** |  |  |  | 700-2508 | 464-1721 | 135-852 | 55-120 | 82-594 |  |  |  | 92–515 |  |  |  |  |

All cell counts in x10E6 cells / L. Reference values: [1]. The age reported is at the time of collecting the blood sample.

NA = Not Available

**Reference**

[1] P.A. Apoil, B. Puissant-Lubrano, N. Congy-Jolivet, M. Peres, J. Tkaczuk, F. Roubinet, and A. Blancher, Reference values for T, B and NK human lymphocyte subpopulations in adults. Data Brief 12 (2017) 400-404.

**Supplemental Table 2. Specifications of fluorochrome conjugated monoclonal antibodies used for spectral flowcytometry in alphabetical order.**


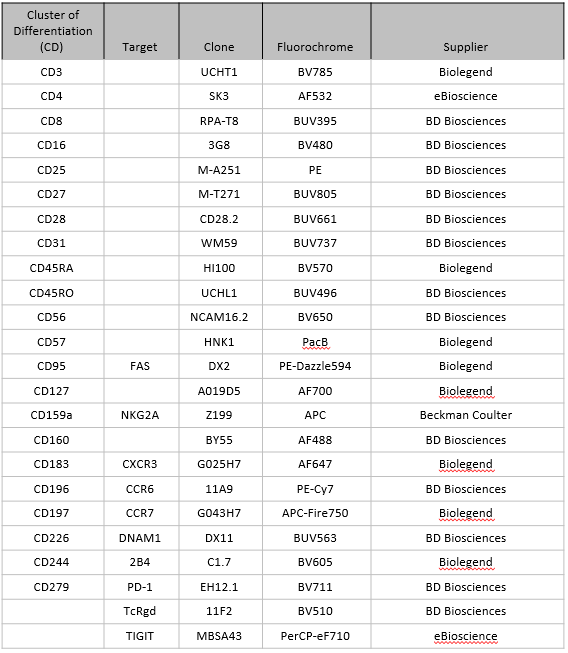


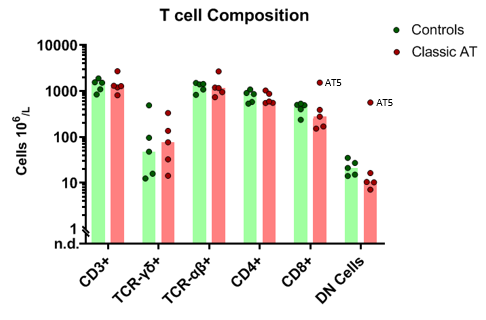


**Supplemental Figure 1. General T Cell Composition.** Bar chart and individual dot plots of the T cell subsets in Classic AT (red) and controls (green). In our small cohort, the absolute numbers of T cells are comparable to controls and mostly in normal range (see also Table S1). Patient AT5 is highlighted as the outlier in the CD8^+^ and DN T cells.


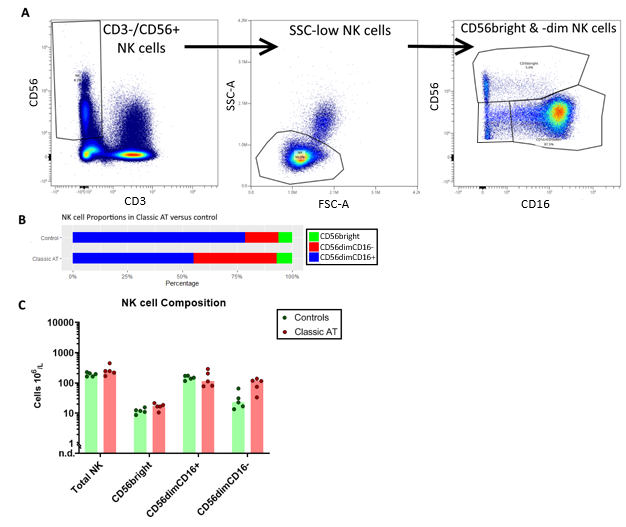


**Supplemental Figure 2. NK cell composition**. **A.** Natural Killer (NK) cells were defined as live, single cell CD3^-^CD56^+^ cells, with low side scatter to exclude monocytes (left and middle plot). Within the NK cells, further subpopulations were defined based on CD56 and CD16; CD56^bright^, CD56^dim^CD16^-^ and CD56^dim^CD16^+^. **B.** Stacked barchart showing the proportions of CD56^bright^, CD56^dim^CD16^-^ and CD56^dim^CD16^+^ cells within the total NK cells. The NK cells are slightly skewed towards less CD56^dim^CD16^+^ cells in Classic AT (bottom) compared to control (top), and more CD56^dim^CD16^-^ NK cells. **C.** The absolute counts of the NK cell subsets, however, as shown in this bar chart and individual dot plots, are comparable between Classic AT (red) and controls (green).


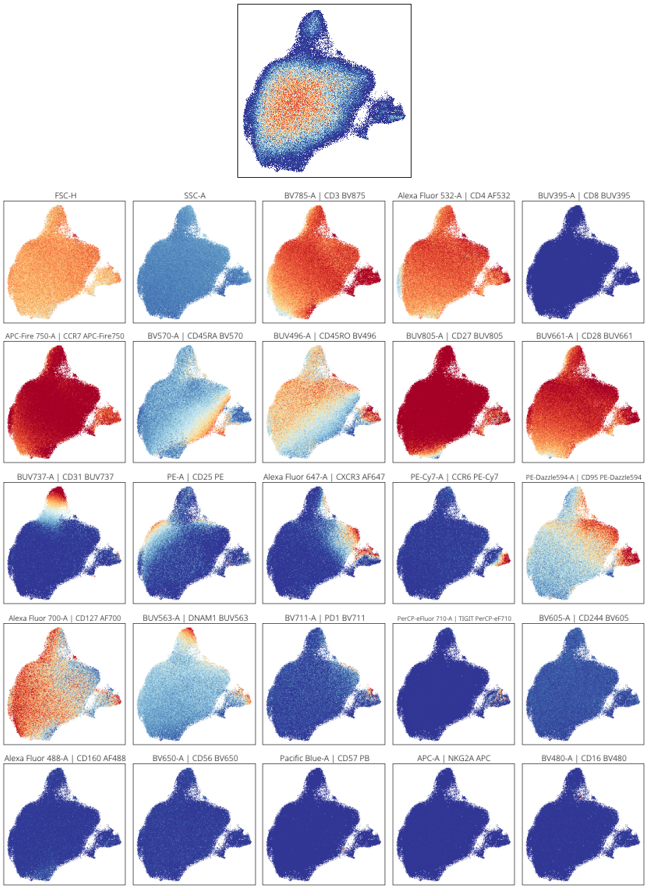


**Supplemental Figure 3. Phenotypical characterization of CD4^+^ CD45RA^dim^CCR7^+^ population in AT1 by UMAP.** As described in the main text and in **Figure 2**. Classic AT sample AT1 has an aberrant CD4^+^ CD45RA^dim^CCR7^+^ population. The cells mostly cluster in one large dense mass, as seen in the top density plot. The marker intensity plots below show mostly homogeneous marker expressions (except for CD25, CXCR3, CD95, CD45RA and CD45RO).


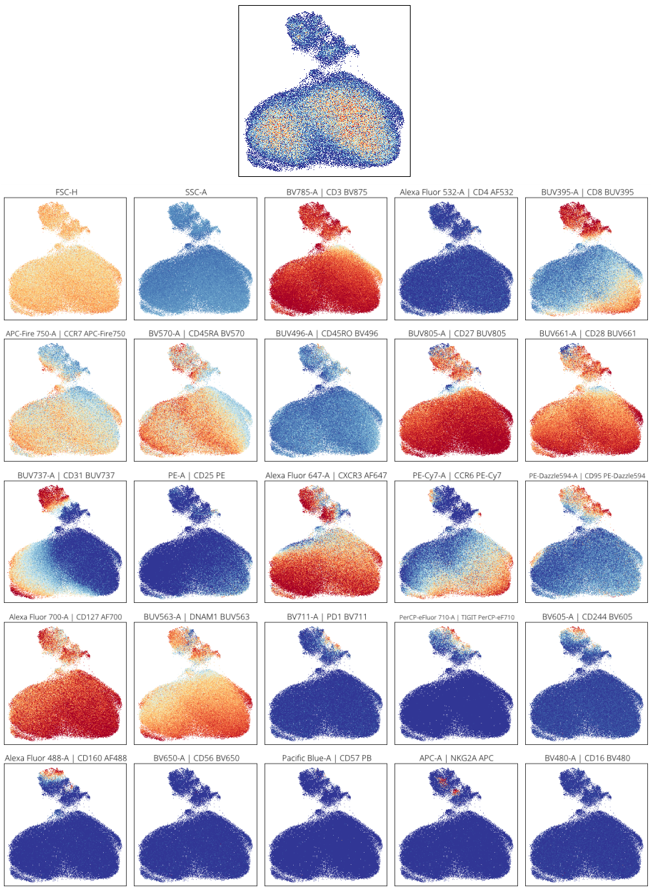


**Supplemental Figure 4. Phenotypical characterization of the CD45RA^dim^CCR7^+^ population within the CD8^+^ gate of AT5 by UMAP.** As described in the main text and in **Figure 2**. Classic AT sample AT5 has an aberrant CD8^+^ CD45RA^dim^CCR7^+^ population. The cells mostly cluster in one large dense mass, as seen in the top density plot. The marker intensity plots below show mostly homogeneous marker expressions (except for CD8, CD31, CCR6).


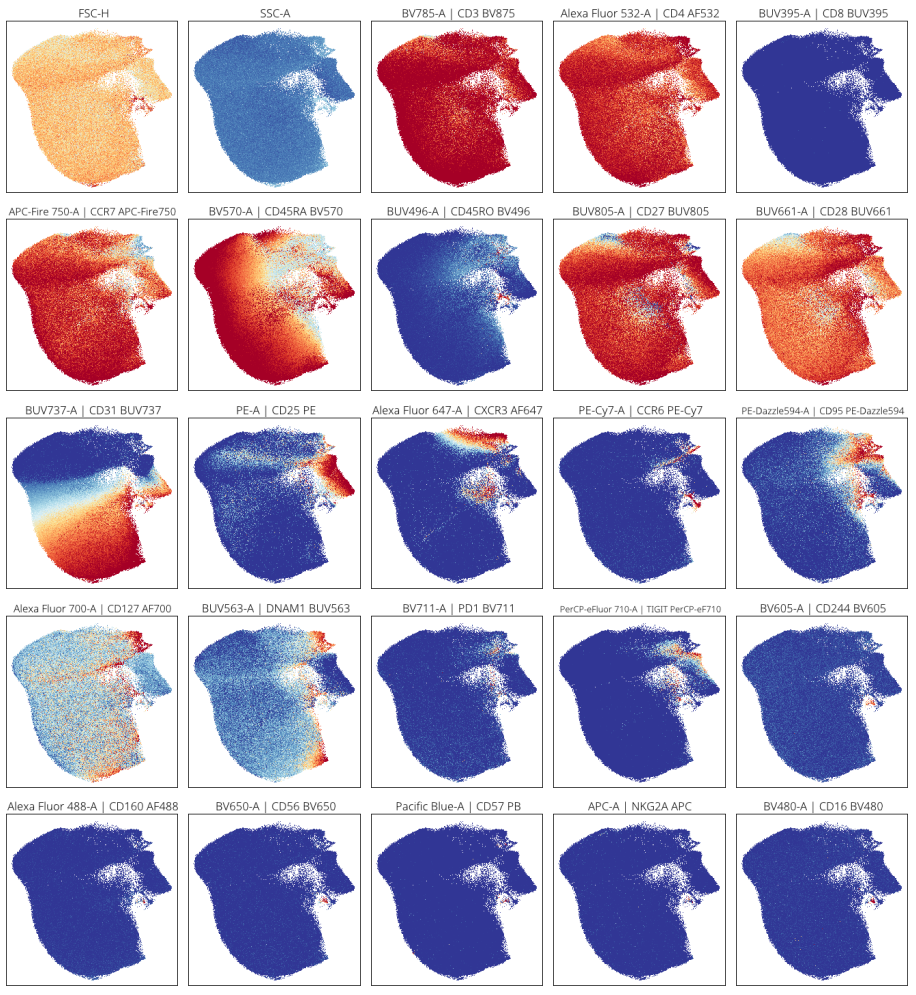


**Supplemental Figure S5A. Phenotype of CD4 naive T cells of the controls.**All individual markers and forward and sideward scatter intensities are overlaid on the UMAP embedding.


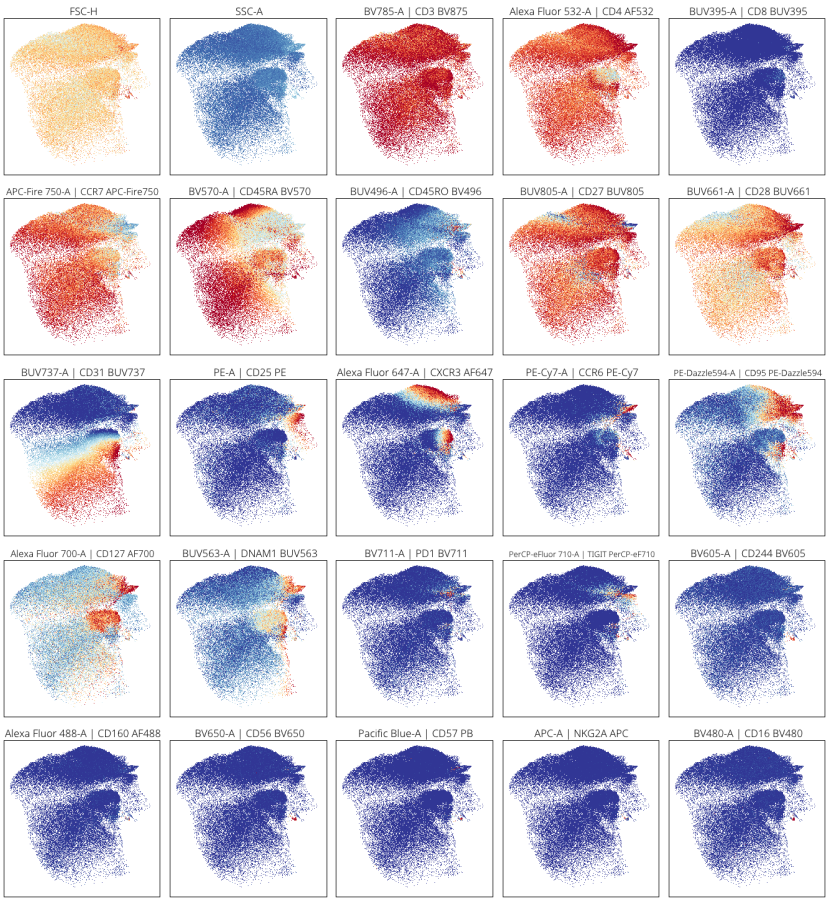


**Supplemental Figure S5B. Phenotype of CD4 naive T cells of Classic AT patients.** All individual markers and forward and sideward scatter intensities are overlaid on the UMAP embedding.


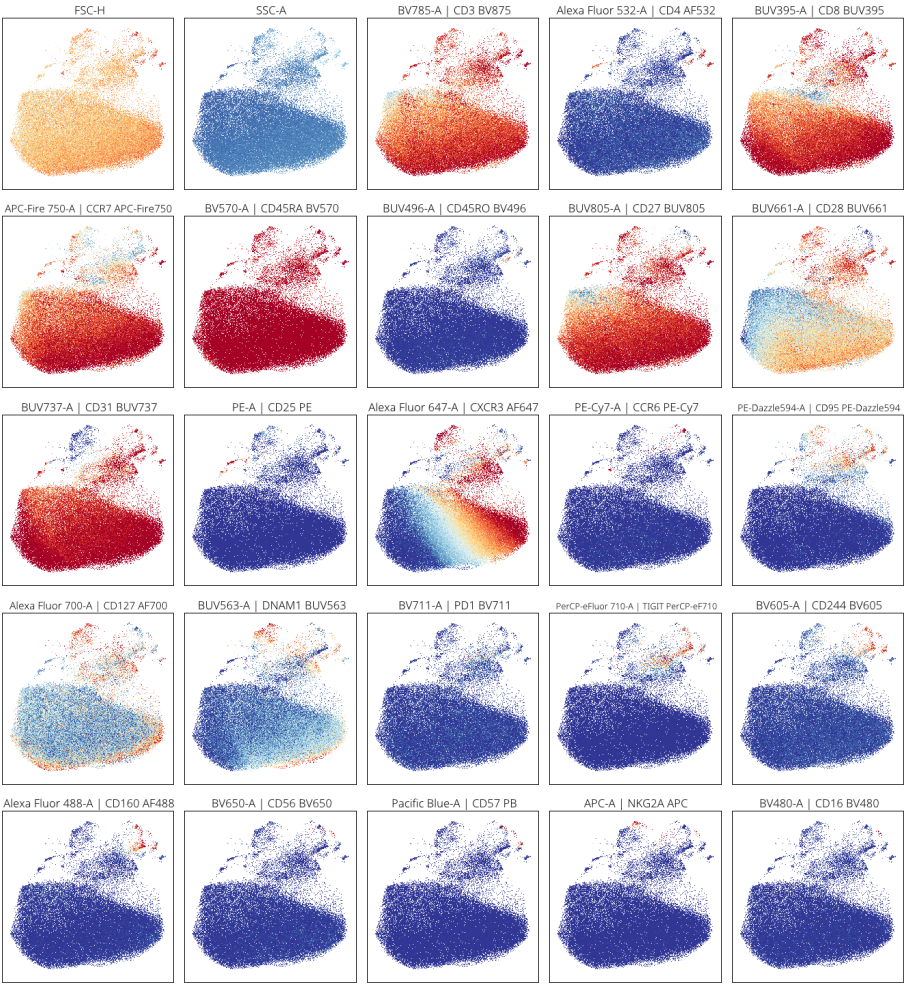


**Supplemental Figure 6A. Phenotype of CD8 naive T cells of the controls.**All individual markers and forward and sideward scatter intensities are overlaid on the UMAP embedding.


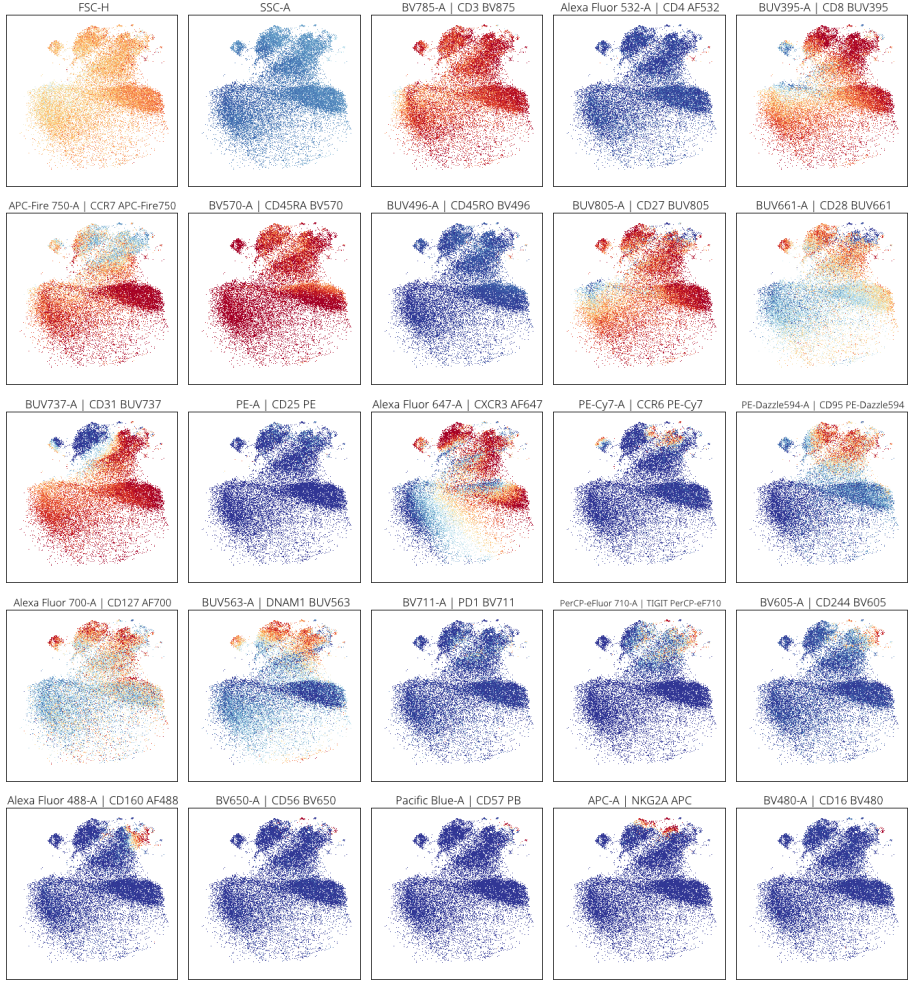


**Supplemental Figure 6B. Phenotype of CD8 naive T cells of Classic AT patients.** All individual markers and forward and sideward scatter intensities are overlaid on the UMAP embedding.


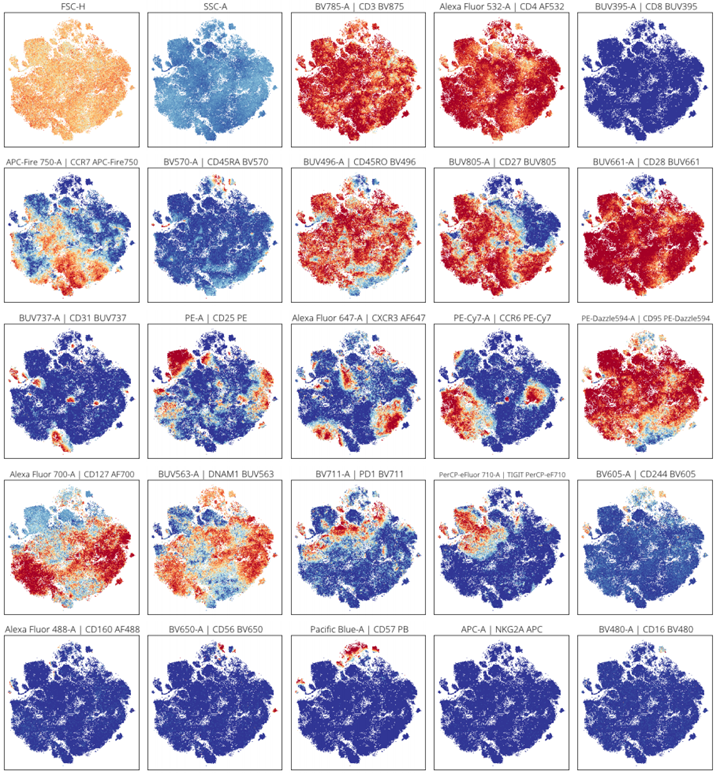


**Supplemental Figure 7A. Phenotype of CD4 Memory T cells of the controls.**All individual markers and forward and sideward scatter intensities are overlaid on the Opt-SNE embedding.


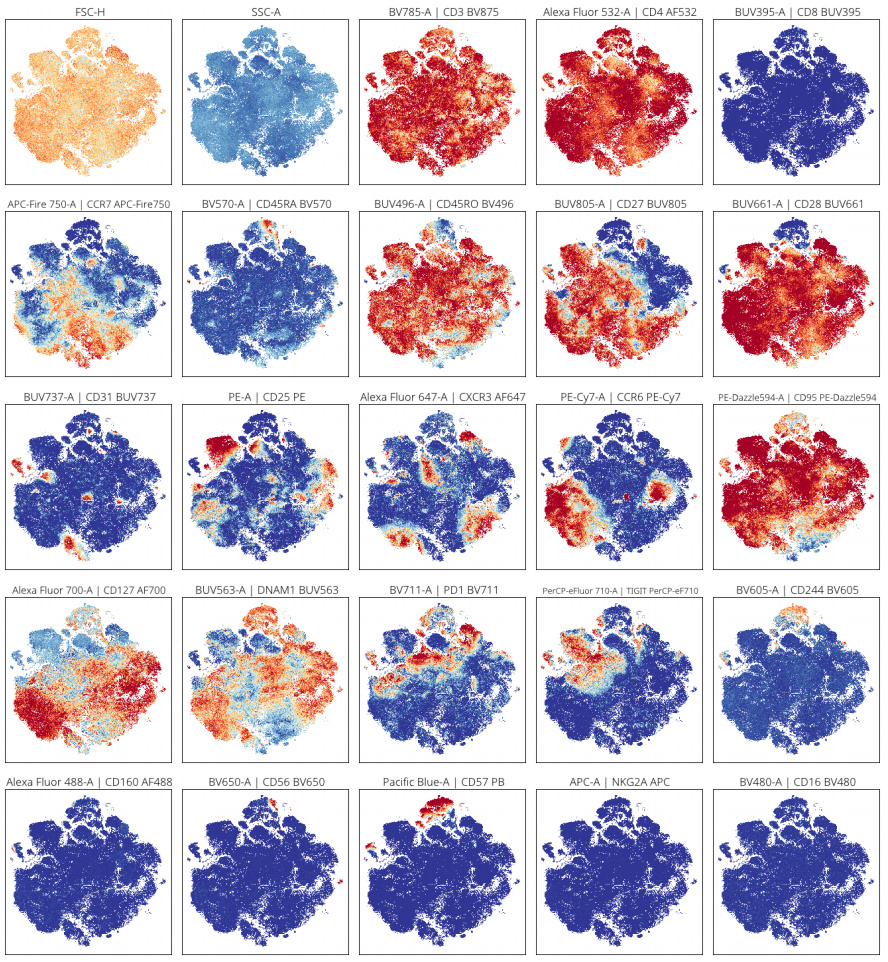


**Supplemental Figure 7B. Phenotype of CD4 Memory T cells of Classic AT patients.**All individual markers and forward and sideward scatter intensities are overlaid on the Opt-SNE embedding.


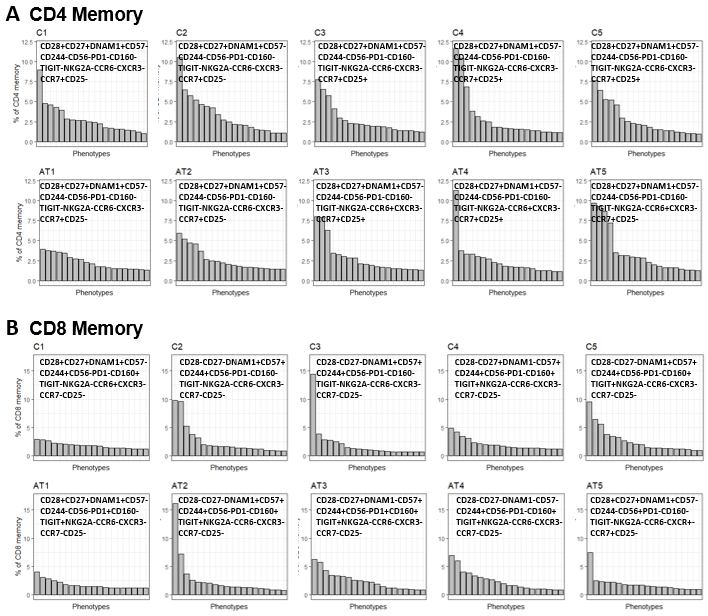


**Supplemental Figure 8. Top 20 most frequent phenotype per sample in CD4 and CD8 memory T cells.** The Boolean gating of the following markers: CD28, CD27, DNAM1, CD57, CD244, CD56, PD1, CD160, TIGIT, NKG2A, CCR6, CXCR3, CCR7 and CD25 resulted in total 16384 possible phenotypes of which 1518 were present in the CD4 memory T cells, and 3396 in the CD8 memory T cells. The distribution of the top 20 most frequent phenotypes in the A) CD4 memory and B) CD8 memory T cells are shown. The most frequent phenotype is shown at the right top.


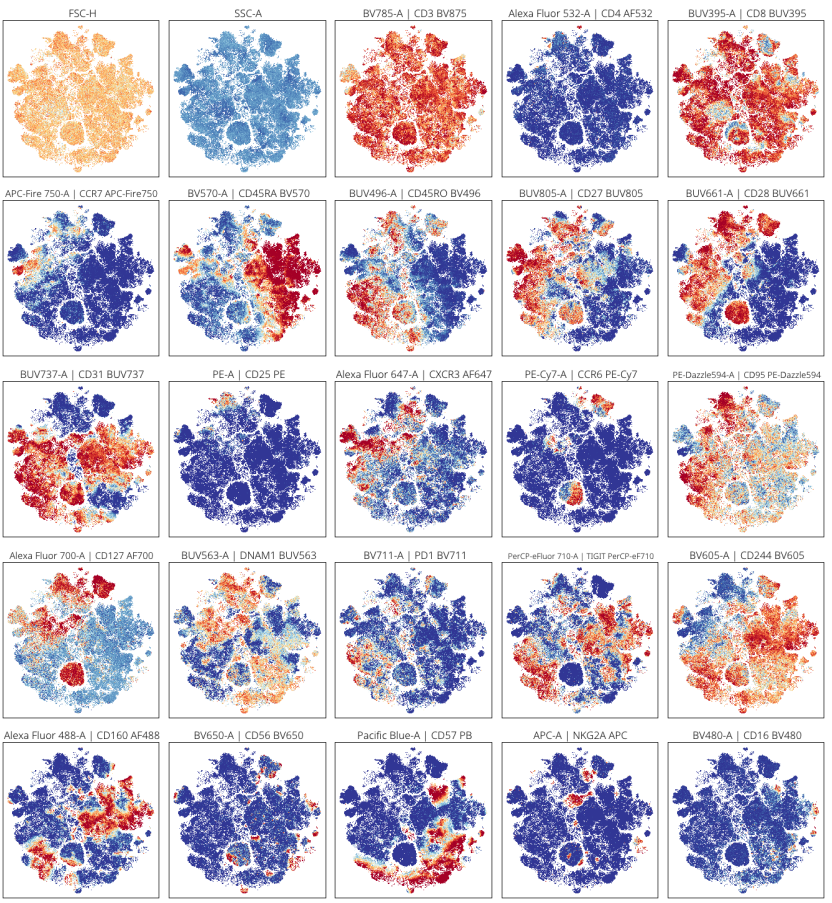


**Supplemental Figure 9A. Phenotype of CD8 Memory T cells of the controls.**All individual markers and forward and sideward scatter intensities are overlaid on the Opt-SNE embedding.


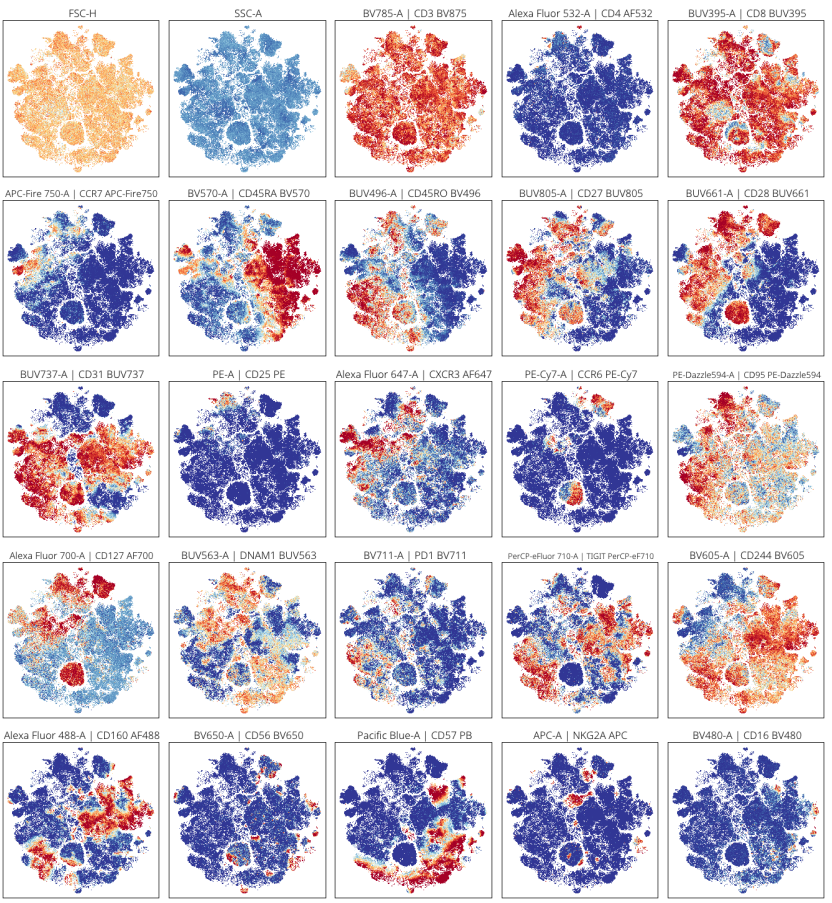


**Supplemental Figure 9B. Phenotype of CD8 Memory T cells of Classic AT patients.** All individual markers and forward and sideward scatter intensities are overlaid on the Opt-SNE embedding.
